# Apathy symptoms modulate motivational decision making on the Iowa gambling task

**DOI:** 10.1186/1744-9081-8-63

**Published:** 2012-12-27

**Authors:** Progress Njomboro, Shoumitro Deb, Glyn W Humphreys

**Affiliations:** 1 Psychology Department, University of Cape Town, Main Road, Rondebosch, Cape Town 7701, South Africa; 2 School of Psychology, Division of Neurosciences, Burlington Danes Building, Imperial College, The Hammersmith Hospital, Du Cane Road, London, W12 0NN, UK; 3 Department of Experimental Psychology, Oxford University, Oxford, UK

**Keywords:** Apathy, Motivational decision making, Iowa gambling task

## Abstract

**Background:**

The present study represents an initial attempt to assess the role of apathy in motivated decision making on the Iowa Gambling Task. Clinical descriptions of patients with apathy highlight deficits in the cognitive, emotional and behavioural aspects of goal directed activity, yet standard neurocognitive tests of these measures fail to demonstrate reliable sensitivity to the disorder. Available research suggests the Iowa Gambling Task is a robust test of complex emotional socio-executive processes involved in motivational decision making, which can analogue real-world goal-directed behaviour.

**Methods:**

We ask whether performance on the Iowa Gambling Task can distinguish brain damaged patients with apathy symptoms from 1) brain damaged patients without apathy and 2) neurologically intact controls. Overall, 22 healthy adults and 29 brain damaged patients took part in this study.

**Results:**

Brain damaged patients with apathy were distinctively impaired on the Iowa Gambling Task compared to both non-apathetic brain damaged patients and neurologically intact healthy controls. On the other hand, standard measures for the cognitive control of behaviour failed to show this sensitivity.

**Conclusions:**

Our results demonstrated that the Iowa Gambling Task is sensitive to the presence of apathy symptoms. We discuss these findings in terms of neurocognition deficits in apathy and the related implications for rehabilitation and clinical intervention.

## Background

Apathy as a syndrome manifests as reductions in motivation, goal directed thoughts, emotions, and behaviour [1]. The disorder is of frequent occurrence following neurological change [2]. Rehabilitation outcome studies on chronic patients going beyond 2 years post brain injury show that the majority of cases experience persisting apathy symptoms with related psychosocial problems characterised by lack of motivation, attenuated emotionality, decreased social contact and leisure activity, unemployment, marital problems and family breakdowns [3]. Apathy is also associated with significant caregiver distress and early institutionalization [4]. Despite these serious clinical implications, the nosological position of apathy and its associated neurocognitive profile remain poorly understood and appreciated in clinical practice [1].

Neurological models suggest apathy follows dysfunction in frontal-subcortical brain circuits crucial for motivation-related executive processing [5,6]. The classic case of Phineas Gage provides the earliest documented case of such dysfunction. Despite his preserved capacities on basic cognition after his brain injury, Gage had significant socio-executive deficits including personality change and apparent apathy to his symptoms [7]. Similar cases have been reported elsewhere, e.g., [8-11]. Apathy is often conceptualised as a dysexecutive syndrome [12], although studies have shown inconsistent results on the relationship between apathy and executive deficits assessed through standard executive function (EF) tests. Some studies have reported an association between apathy symptoms and poor performance on these tests [13,14] while others have found no such an association. For instance, a review by van Reekum and associates [15] found a near middle split in the number of studies that reported a significant relationship between apathy levels and executive deficits (8 studies) and those that did not (7 studies).

Use of different tests and scales on different clinical samples across studies may partly account for the mixed results. Also, in some cases real-life dysexecutive behaviour has been seen to dissociate from deficits on standard EF tests [16-18]. Patients with significant functional problems often perform relatively well on these ‘offline’ tests [19]. Robust tasks that are sensitive to real life socio-executive processing may help us understand the nature of neurocognitive impairment in apathy. The socio-executive processing needed for successful Iowa Gambling Task (IGT) performance is thought to analogue real life motivated behaviour [20,21], and therefore makes a potentially useful test for the deficits in goal directed behaviour that comprise apathy symptoms.

A number of reasons make the IGT a plausible test for apathy symptoms. Firstly, brain areas thought to subserve emotional-executive processes crucial for effective IGT performance such as the medial frontal regions, anterior cingulate cortex, and amygdala form part of the cortico-subcortical neural circuitry responsible for motivated goal direct behaviour [22-28]. Damage to these areas reliably causes apathy [29-35]. Secondly, apathy and impaired IGT performance have both been linked to malfunctions of the dopamine system. Blockade of dopamine has been reported to impair, and stimulation of dopamine to improve, IGT performance [36]. Enhanced IGT performance due to stimulation of dopamine is also consistent with the view that dopamine mediates exploratory behaviour and the suggested effectiveness of dopamine agonists in combating apathy symptoms [37,38]. With this converging evidence, the IGT provides an attractive premise to build a theoretical framework for understanding neurocognitive correlates of apathy symptoms.

In this study we assessed whether the IGT is sensitive to the presence of apathy symptoms in patients with acquired brain damage. It is also possible that the presence of apathy symptoms may impair performance on any test as a result of general motivational deficits. For that reason, we also obtained independent EF measures on the Brixton Spatial Anticipation test [39].

## Method

### Participants

In total 51 participants took part in the study. Twenty nine brain damaged patients were recruited from brain injury clinics in the West Midlands region of England. Most neurocognitive studies on apathy have been done on homogenous patient samples, thus making it difficult to isolate the influence of the aetiological process, apathy symptoms, and executive processing deficits. To increase the power of the relationship between apathy symptoms and socio-executive deficits, we profiled brain damaged patients from a number of aetiologies (Cerebrovascular accident = 10; Head injury = 7; Anoxia = 5; Herpes Simplex Encephalitis = 5; Aneurysm = 2). For this reason, the patients’ lesions were also of varied locations. Lesion location data for each of the patients was obtained from brain scan information in the patients’ clinic files (See Table 1 for the lesion data).

**Table 1:** Lesion location and apathy diagnosis

***Lesion***	***IAES - 41 score cut off***		***Total***
	***Apathy***	***No apathy***	
Left parietal	2	2	4
Right parietal	1	2	3
Bilateral parietal	1	0	1
Left fronto-temporal	1	3	4
Right fronto-temporal	3	1	7
Bilateral fronto-temporal	10	2	12
*Total (N)*	18	10	28

Twenty-two healthy adult controls recruited through local adverts in the West Midlands city of Birmingham also provided the normative data on the IGT (See Table 2 for the participants’ demographic characteristics). All participants gave informed written consent to participate in the study. Ethics approval for the study was obtained from the Birmingham and Solihull Research Ethics Committee.

**Table 2:** Participants age and education characteristics

		***N***	***Mean***	***SD***	***Sig.***
*Age*	Control	22 (M=14; F=8)	44.00	18.60	p > .05
	No Apathy	10 (M=7; F=3)	56.50	14.17	
	Apathy	18 (M=18; F=1)	53.06	14.04	
*Education*	Control	22 (M=14; F=8)	14.91	1.02	p > .05
	No Apathy	10 (M=7; F=3)	14.40	0.84	
	Apathy	18 (M=18; F=1)	14.53	.90	

### Apathy symptoms

The informant version of the Apathy Evaluation Scale (AES-I) [40] was used to assess apathy. The AES-I is an 18-item scale that assesses behavioural, emotional, and cognitive aspects of apathy. Each item, *(e.g., s/he gets things done during the day)* is rated on a scale of 1 *(Not at all characteristic)* to 4 *(A lot characteristic)*. The scale has enjoyed widespread use and has good psychometric properties [40]. Patients’ caregivers provided the evaluations. We took apathy scores of 41+ as indicative of the presence of apathy (supplementary administration and scoring guidelines obtained from the authors). To control for the likely influence of depression on the relationships between study variables, the Beck Depression Inventory (BDI; [41]) was also used to evaluate levels of depressive symptoms in the patient samples.

### Executive function measures: the Brixton test

The Brixton Spatial Anticipation test [39] provided standard EF measures. This test was chosen because of its robustness and sensitivity to a variety of executive deficits, including preservative behaviour and failure to utilise feedback or follow rules [39].

### Socio-executive measure: the Iowa gambling task

The IGT requires participants to choose cards from four decks labelled A, B, C, and D. Each deck is made up of 40 cards. The task is rigged such that cards from decks A and B give high rewards (£100) on each card selection, but also yield unpredictable and large losses such that continuously picking from these decks results in a net loss. Decks A and B are therefore risky decks. On the other hand, Decks C and D are safe. They give relatively lower immediate gains on each picking (£50), but the associated losses are also lower. Picking more cards from the safe decks C and D gives a net gain, and picking more cards from the risky decks A and B results in a net loss.

Because we used the non-automated card version of the IGT, some of the patients ran out of play money at various points after the 3^rd^ Block (see below). Also, because the non-automated IGT version has fixed numbers of cards for each deck, better performers ran out of safe picks before reaching the final round of choices and had to pick from the risky decks out of lack of choice. For these reasons, we only analysed card choices up to the 60^th^ round. While this is a potential limitation to our study, it is important to note that in neurologically intact subjects, the trend towards avoiding risky decks is often set by the 40th choice [21].

## Results

Tests for sphericity and homogeneity of variance were performed on the data to determine whether it met the assumptions for the use of parametric tests. Our data met these assumptions.

### Apathy evaluation

Nineteen patients (Male = 18, Female = 1; Mean age = 54, SD = 14.25) met criteria for the presence of apathy (Mean apathy scores = 52.8, SD = 7.4) and 10 patients (Male = 7; Female = 3; Mean age 56.5, SD = 14.17) did not have apathy (Mean = 33.2, SD = 5.5).

### Gambling task

**Figure 1:**
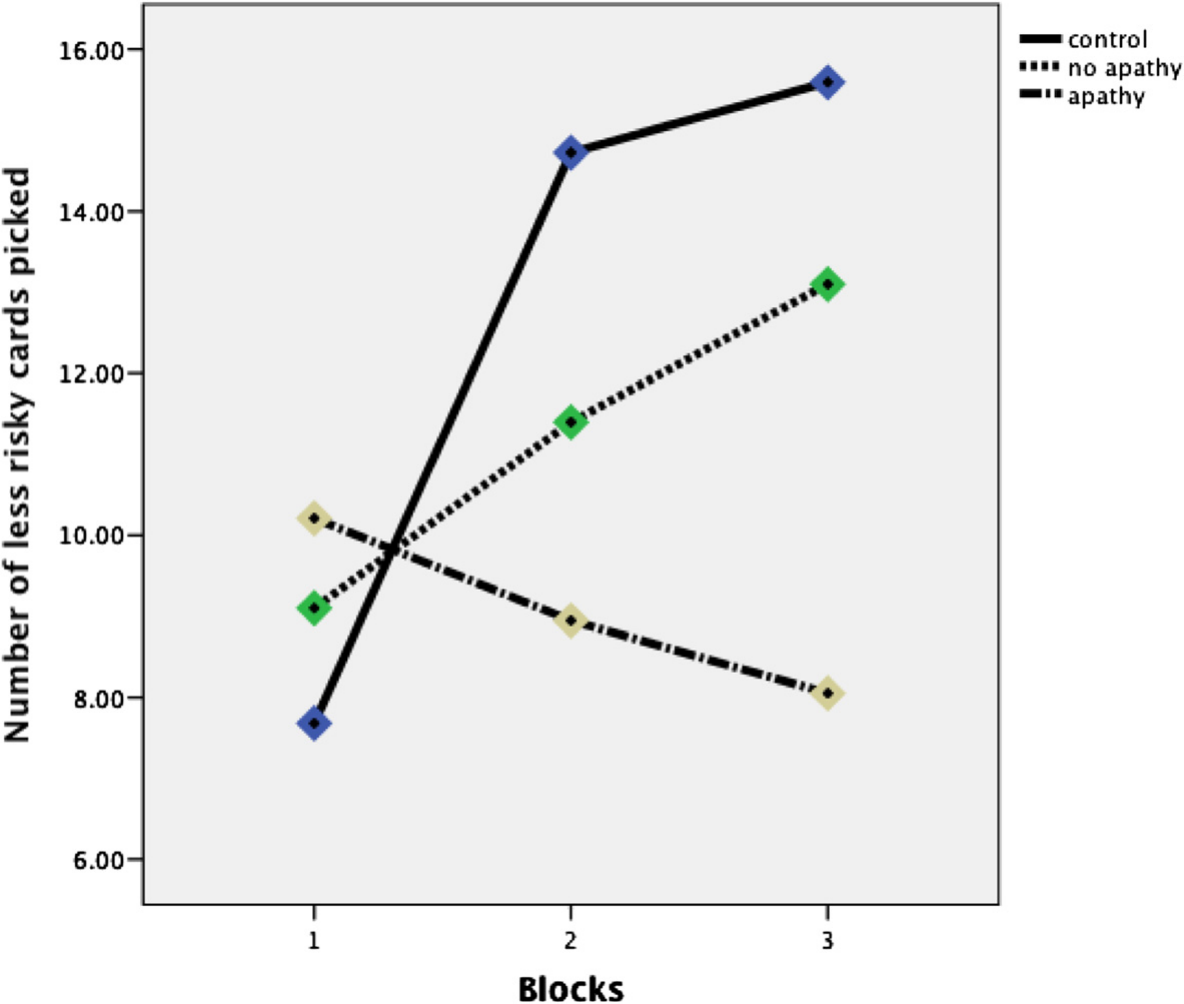
The three graphs show less risky card selections for patients with apathy symptoms, patients without apathy and health controls across Blocks 1, 2 and 3.

To investigate the participants’ gambling trends, we divided the rounds of choices into 3 blocks: Block 1 (rounds 1–20), Block 2 (rounds 21–40), and Block 3 (rounds 41–60). Neurologically intact controls picked fewer safe cards (from decks C and D) in Block 1 (Mean = 7.68, SD = 2.81); they then picked more safe cards in Block 2 (Mean = 14.73, SD = 3.8) and even more safe cards in Block 3 (Mean = 15.59, SD = 3.54). The same trend towards picking more from the safe C and D decks as the game progressed was also observed for non-apathetic patients (Block 1: Mean = 9.1, SD = 3.54; Block 2: Mean = 11.40, SD = 4.69; and Block 3, Mean = 13.10, SD = 6.0). In contrast, although on average apathetic patients picked more safe cards in block 1 compared to the other two groups (Mean = 10.21, SD = 3.46), they eventually picked fewer safe cards in block 2 (Mean = 8.95, SD = 4.43) and even fewer safe cards in block 3 (Mean = 8.05, SD = 4.97). Figure 1 shows the mount of safe picks (from decks C and D) for the 3 participant groups.

A mixed ANOVA performed on the safe (C+D) scores for the 3 participant groups with Block (Blocks 1, 2, and 3) as a within subject factor and Participant Type (Normal, Non-apathetic, and Apathetic) as a between subjects variable showed significant main effects of Block F(2, 98) = 31.10, p < .001, r = .24. The interaction between Block and participant type was also significant F (2, 98) = 21.08, p < .001, r = .39. When depressive symptoms (BDI score) and executive function deficits (Brixton test scores) were controlled for as covariates for the two patient groups, there where non-significant effects of executive function deficits F (1, 21) = 1.02, p > .05, r = .22, and of depressive symptoms, F (1, 21) = .004, p > .05 r = .1. The effects of Block remained significant, F (1, 21) = 3.81, p < .05 r = .45.

Further one way ANOVA tests on risky gambling scores showed significant group differences (F (2, 48) = 9.20, p < .001 r = .41). Pairwise comparisons indicated that apathetic patients made significantly more risky card choices compared to both normal controls (Mean difference = 9.9; p < .001, r = .28) and patients without apathy (Mean difference = 5.44; p < .05, r = .18). IGT scores for the non apathetic patients were not significantly different from scores for healthy controls (Mean difference = 4.1; p > .05, r = .26). See Table 3 for these comparisons.

**Table 3:** Pairwise comparisons on gambling task performance between apathetic patients, non-apathetic patients, and healthy controls

	***Mean Difference***	***Std Error***	***Significance***
Apathetic vs. Non-apathetic	5.44	1.05	p < 0.05
Apathetic vs. Normal controls	9.9	0.84	p < 0.001
Non-apathetic vs. Normal controls	4.1	1.02	p > 0.05

**Figure 2:**
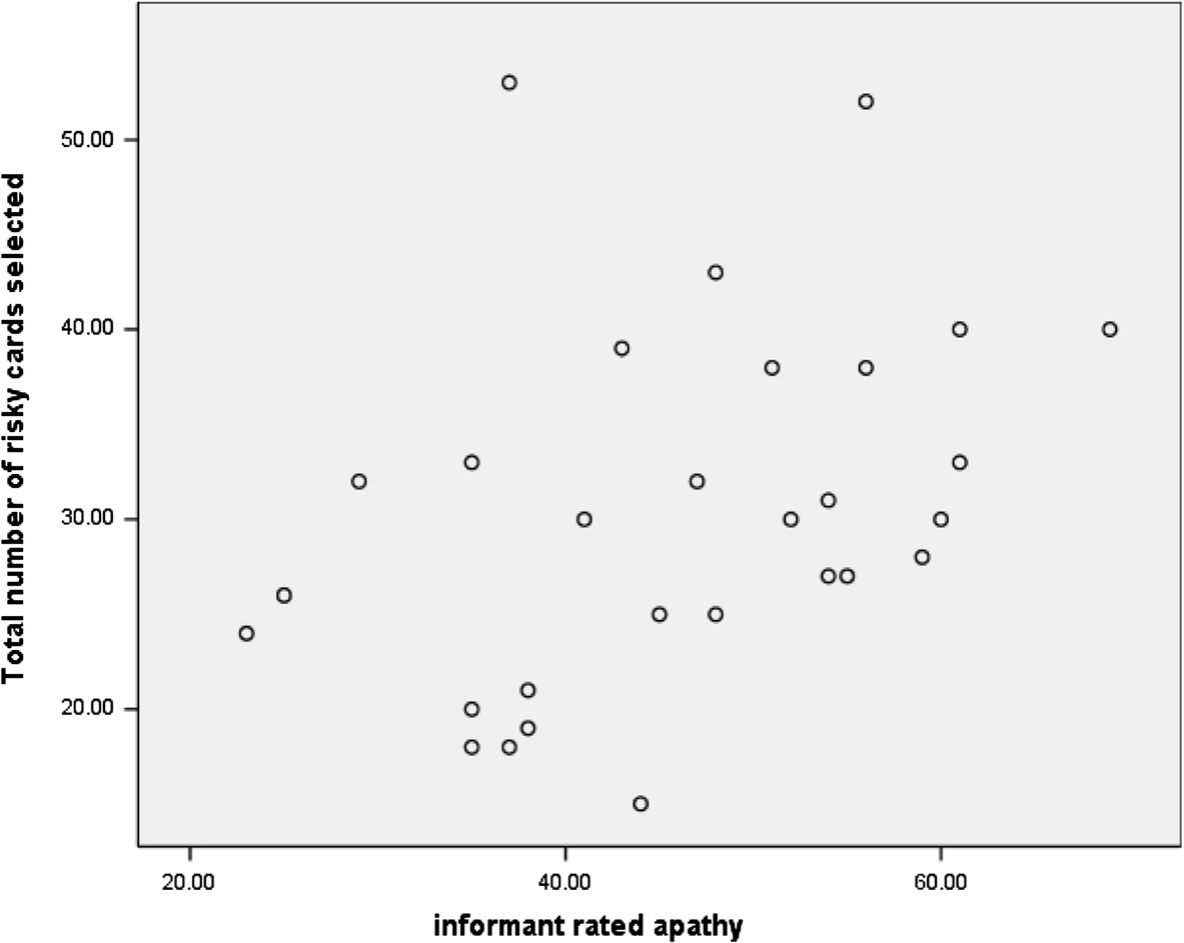
The relationship between levels of apathy and the number of risky card selections.

There is little consensus on the appropriate cut-off score for the AES-I. Cut-off scores have ranged from 35 to 41 across studies (Glenn et al., 2002; Lane-Brown & Tate; 2009). For this reason we also performed Pearson correlation analyses between patients’ AES-I apathy ratings and the total number of risky cards they selected. The results showed a significant positive relationship between the level of apathy symptoms and the number of risky cards selected, r = .38, p < 0.05. Figure 2 shows the scatter plot for this relationship and illustrates the point that patients with high levels of apathy also picked relatively more risky cards.

## Discussion

Patients with apathy made significantly more risky choices on the IGT compared to neurologically intact and brain damaged non-apathetic controls, both of whom (as groups) picked less from the risky decks as the game progressed. The shift from risky to the safe decks shown by healthy participants is in line with the performance patterns reported in similar studies [42-47]. Non-apathetic brain damaged patients also shifted towards good decks as the game progressed.

It is possible that apathetic patients may perform poorly due to their general lack of motivation, and not necessarily due to a specific cognitive deficit related to the particular task. This argument is however inconsistent with our findings. For instance, putting in the Brixton test score as a covariate did not dilute the effect, suggesting the two patient groups’ performance on this test was not significantly different. We can claim with some degree of confidence that the IGT is sensitive to the presence of apathy symptoms, and distinguished apathetic from non-apathetic patients (and normal controls).

The IGT’s sensitivity to apathy symptoms makes it a potentially valuable instrument for both research and clinical practice. In a number of cases standard off-line EF measures fail to distinguish between patients with real-life socio-executive deficits like apathy and those without these deficits [48]. Standard EF tests may provide test takers with reliable task instructions and the type of feedback that is not found in more complex real life scenarios. On the other hand, the IGT has open ended behavioural choices and requires the generation of self-initiated choices under less explicit feedback. Such conditions provide a more valid analogue of socio-executive demands found in real life social environments. Our results suggest that apathy symptoms may arise from specific deficits in a patient’s capacity to produce or structure their own goal directed behaviour. This is in line with the realisation that apathetic patients rely heavily on others to structure their own activities [49]. Rehabilitation programmes in which daily tasks are structured, with enough prompts and cues may benefit patients with apathy. In this context, the IGT can be a useful addition to assessment batteries for detecting socio-executive deficits related to apathy.

Effective IGT performance is thought to depend on intact emotional processing [50]. According to this view (the somatic marker hypothesis), effective IGT performance benefits from subconscious emotional capacities that pre-bias responses away from bad behavioural choices. This bias enhances the selection and efficient execution of good behavioural choices, and is thought to underlie adaptive goal-directed activity, reasoning and decision-making in real life social contexts. It is tempting to suggest an inability to develop or fully utilise such emotional biasing signals to explain apathy symptoms. Empirical studies support this viewpoint. For instance, it has been observed that patients with apathy show deficits in matching emotional responses to social situations [51,52]. Furthermore, there is evidence for a significant inverse association between apathy and autonomic excitation measured through heart rate reactivity [53]. Our study is however limited in the extent to which we can ascribe poor performance on the IGT to such lack of autonomic emotional inputs because we did not obtain concurrent measures of autonomic arousal.

The IGT’s sensitivity to apathy symptoms may also lie in its requirement for sensitivity to rewards and punishments. Part of the apathy symptom profile includes a dimension of indifference to rewards and punishments, lack of interest or concern, anhedonia, and social disengagement [54,55]. Apathetic patients may have picked more risky cards due to insensitivity to punishments. Bechara and colleagues [56] have however demonstrated that sensitivity to rewards or insensitivity to punishment contingencies is not enough to explain impaired IGT performance. They either reversed rewards and punishments on the task such that good decks yielded higher immediate punishments but even greater delayed rewards, while risky decks gave off low immediate punishments and even lower future rewards, and in another condition increased the future disadvantages on risky decks. Patients with dysexecutive deficits preferred decks that had low immediate punishment to those with higher immediate punishment but more rewards in the long run. In other words large delayed rewards failed to lure the patients to the paying decks while on the other hand the patients were reluctant to choose from decks with huge initial punishment indicating that hyper sensitivity to rewards or insensitivity to punishments might not be a crucial factor on effective IGT performance.

Fellows and Farah [22] suggest that impaired goal directed behaviour can result from impaired future time perspective, which is a measure of an individual’s self defined future. Interestingly, deficits in future time perspective were found to correlate with self-reported apathy symptoms [22]. Whether this deficit can explain impaired IGT performance in this study is a subject for future research. There are also some suggestions that deficits in reversal learning or inhibition could account for impairments on the IGT. Effective performance requires that participants switch from initially high paying decks to lower paying but also low punishment decks. This involves reversal learning and the inhibition of responses to high reward/high cost decks - capacities that can be impaired in some brain damaged patients [57]. It is unlikely that deficits in reversal learning or inhibition could account for our results though, since apathetic patients were not distinctly impaired on the Brixton test.

Other factors, such as the effects of working memory deficits on IGT performance [58], and the cognitive impenetrability of the task [59,60] have been suggested to explain impaired performance on the task. However, Bechara, et al. [61] have shown that working memory and decision making are dissociable and also shown that the majority of normal participants begin avoiding risky decks before confessing awareness of the game’s rewards and punishments contingencies. See also [62].

The IGT’s main weakness is its lack of specificity. A majority of different patient populations have shown deficits on the measure. Impaired IGT performance has been reported in schizophrenics [63-65]; in pathological gamblers [66]; in alcohol, marijuana, and substance dependent individuals [67-69]; in anorexics [70] in patients with intermittent explosive disorder and conduct disorder [71,72]; in Huntington’s disease sufferers [24]; in traumatic brain injury patients [73]; in frontotemporal dementia [74] and in HIV infected patients [75]. It is nevertheless important to note that apathy is also a common syndrome in most of these disorders [15].

The IGT’s lack of specificity across different neuropsychiatric disorders could also be an indication of the task’s sensitivity to a wider spectrum of deficits, for instance, involving working memory, the patient’s interest and concern about the game, the processing of reward and punishment, explorative behaviour, and capacities related to the inhibition of choices on highly paying but risky decks.

## Conclusions

The IGT’s sensitivity to apathy symptoms makes it a potentially valuable neurocognitive tool for assessing these symptoms. However the future utility of the task rests upon our ability to isolate the potential causes of impaired performance in specific clinical populations and syndromes. This should help unravel the individual aspects of the task that, for instance, give it sensitivity to apathy symptoms. Future studies may try and isolate or control for these various variables that can potentially impair IGT performance. Future studies may also consider using the electronic version of the IGT which allows the administration of the full 100 rounds even with poor or extremely good performers.

## Competing interests

The authors declare that they have no competing interests.

## Authors’ contributions

All authors made significant contributions to the study. All authors read and approved the final manuscript.
